# A real-world disproportionality analysis of cyclosporine from the FDA Adverse Event Reporting System (FAERS) database

**DOI:** 10.1590/1414-431X2024e13392

**Published:** 2024-07-29

**Authors:** Shichao Cui, Li Li, Wensheng Liu, Bin Zhao, Xingming Zhong

**Affiliations:** 1 NHC Key Laboratory of Male Reproduction and Genetics, Guangdong Provincial Reproductive Science Institute (Guangdong Provincial Fertility Hospital), Guangzhou, China; 2 Xiamen Health and Medical Big Data Center, Xiamen, China; 3 Xiamen Medicine Research Institute, Xiamen, China; 4 Xiamen Key Laboratory of Natural Medicine Research and Development, Xiamen, China

**Keywords:** Cyclosporine, Immunosuppressant agent, Pharmacovigilance, Data mining, Serious adverse events, FAERS

## Abstract

Cyclosporine is an immunosuppressant used to prevent organ rejection in kidney, liver, and heart allogeneic transplants. This study aimed to assess the safety of cyclosporine through the analysis of adverse events (AEs) related to cyclosporine in the US Food and Drug Administration Adverse Event Reporting System (FAERS). To detect AEs associated with cyclosporine, a pharmacovigilance analysis was conducted using four algorithms on the FAERS database: reporting odds ratio (ROR), proportional reporting ratio (PRR), Bayesian confidence propagation neural network (BCPNN), and empirical Bayes geometric mean (EBGM). A statistical analysis was performed on data extracted from the FAERS database, covering 19,582 case reports spanning from 2013 to 2022. Among these cases, 3,911 AEs were identified, with 476 linked to cyclosporine as the primary suspected drug. Cyclosporin-induced AEs targeted 27 System Organ Classes (SOCs). Notably, the highest case at the SOC level included eye disorders, injury, poisoning, and procedural complications, as well as immune system disorders, all of which are listed on the cyclosporine label. Furthermore, we discovered novel potential AEs associated with hepatobiliary disorders, among others. Moreover, unexpected adverse drug reactions (ADRs), such as biliary anastomosis complication and spermatozoa progressive motility decrease, were identified. Importantly, these newly identified ADRs were not mentioned on the cyclosporine label, which were involved in injury, poisoning, and procedural complications, and investigations at the SOC level. The study used pharmacovigilance analysis of FAERS database to identify new and unexpected potential ADRs relating to cyclosporine, which can provide safety tips for the safe use of cyclosporine.

## Introduction

Cyclosporine is a cyclic polypeptide immunosuppressant agent consisting of 11 amino acids. It is produced as a metabolite by the fungus species *Tolypocladium inflatum* Gams. This drug was discovered in 1970 and first introduced for prevention of solid organ graft rejection in 1983 (1). In 1995, the U.S. Food and Drug Administration (FDA) approved 0.2% cyclosporin ophthalmic ointment (Optimmune^®^; ScheringPlough, USA) for the treatment of kerato-conjunctivitis sicca (KCS) (2). In 2002, the FDA approved Restasis (0.05% cyclosporine ophthalmic emulsion, Allergan Inc., USA) for tear production increase in people whose tear production is presumed to be suppressed due to ocular inflammation associated with KCS (chronic dry eye) (3). The FDA also approved Cequa (0.09% cyclosporine drops, Sun Pharma, USA) for increase in tear production in 2018 (4).

Because its effect is related to the inhibition of T-lymphocyte-dependent immune response by interfering with the synthesis of interleukin-2 (5,6), the indication of cyclosporine according to the FDA is for the prophylaxis of organ rejection in allogeneic kidney, liver, and heart transplants. It can also be used in the treatment of chronic rejection in patients previously treated with other immunosuppressive agents. According to the Canadian Compendium of Pharmaceuticals and Specialties (CPS) (7), the official Canadian indications for cyclosporine are solid organ transplantation, bone marrow transplantation, psoriasis, rheumatoid arthritis, and nephritic syndrome. According to the literature, the indications of cyclosporine have recently broadened to manage diseases such as psoriasis, rheumatoid arthritis, systemic lupus erythematosus, atopic dermatitis, and myasthenia gravis (8). In addition, research shows that women with a single or repeat liver transplant, under appropriate multidisciplinary care, stable graft function at pregnancy onset, and adherence to immunosuppressive regimens, can have a low rate of graft complications, although they are at increased risk for pregnancy complications. Immunosuppressants, including cyclosporine, can be safely used for maintenance of graft function and management of graft deterioration in pregnancy (9).

The most well-known major adverse events (AEs) of cyclosporine are nephrotoxicity and hepatotoxicity. The incidence of minor adverse reactions such as tremor, paresthesia, malaise, headache, gingival hypertrophy, and hypertrichosis varies between 31 and 51% (10,11). The expanding indications for cyclosporine therapy will surely result in an increasing number of side effects. Therefore, it is important to exercise prudence regarding the potential occurrence of AEs following treatment with cyclosporine, regardless of whether they are explicitly mentioned in the product label or not. The FDA Adverse Event Reporting System (FAERS) database, a public spontaneous reporting system (SRS), contains tens of millions of adverse drug event (ADE) reports from physicians, pharmacists, manufacturers, and others (12). The FAERS is currently the world's largest pharmacovigilance database and is an effective tool for searching information related to adverse drug reactions (ADRs). In the present study, we aimed to evaluate AEs of cyclosporine through FAERS data mining to provide a reference for its clinical monitoring and risk identification.

## Material and Methods

### Data source and preprocess

We conducted a retrospective pharmacovigilance analysis using FAERS data from the first quarter of 2013 to the fourth quarter of 2022 (the study's most recent FAERS database update). The data from the FDA website was imported into MySQL 8.0 (https://www.mysql.com/) in order to facilitate analysis for future examination. The collected data was cleaned of duplicated case records. This was achieved by coding the individual safety report (ISR) and mapping the drug names to RxNorm concepts, as well as ADR outcomes to Medical Dictionary for Regulatory Activities (MedDRA^®^) concepts (13). There are four levels of terminology in MedDRA: the System Organ Class (SOC), the high level group term (HLGT), the high level term (HLT), and the preferred term (PT). As required by the US FDA (14), the SOC and PT were used as analytical elements of case reports. The research collected AEs linked to cyclosporine over a ten-year period. The investigators classified the principal clinical outcomes into four categories: death, disability, hospitalization, and life-threatening conditions. Additionally, the study considered supplementary factors such as gender, age, and nationality.

### Signals detection

The FAERS database is a spontaneous reporting system. Therefore, the total number of individuals exposed to the drug of interest in a given time span was difficult to estimate. Pharmacovigilance studies employed the technique of disproportionality analysis, which is a fundamental method within analytical methodologies. This approach is utilized to ascertain the relative occurrence of AEs associated with a specific medication compared to all other pharmaceuticals. Various statistical methods, including the reporting odds ratio (ROR), proportional reporting ratio (PRR) (15), Bayesian confidence propagation neural network (BCPNN), and empirical Bayes geometric mean (EBGM), are employed for this purpose (16).

In order to compute the ROR and PRR, the values of variables a, b, c, and d, as shown in Table 1, were computed. Variable ‘a' represents the number of individuals who experienced a specific adverse event following exposure to cyclosporine. Similarly, variable ‘b' represents the number of individuals who experienced adverse events unrelated to cyclosporine exposure. Variable ‘c' represents the number of individuals who encountered the specific adverse event after non-cyclosporine drug exposure. Variable ‘d' represents the number of individuals who experienced the non-specific adverse event after exposure to non-cyclosporine drug. Formulas for each of the four algorithms are listed below:

- ROR algorithm: $ROR=(ad)/(bc);\;95\%\;CI=e^{\ln(ROR)\pm 1.96\sqrt{\frac{1}{a}+\frac{1}{b}+\frac{1}{c}+\frac{1}{d}}}$ (positive safety signals are detected by the lower limit of 95%CI > 1, N ≥ 3);
- PRR algorithm: $PRR=\frac{\left[a(c+d)\right]}{\left[c(a+b)\right]};\;\chi^{2}=\frac{(a+b+c+d){\left(ad-bc\right)}^{2}}{\left(a+b)(c+d)(a+c)(b+d\right)}$ (standards for detecting positive safety signals: PRR ≥ 2, χ^2^ ≥ 4, N ≥ 3);
- BPCNN algorithm: $IC=\log_{2}\frac{a(a+b+c+d)}{\left(a+b)(a+c\right)};\;95\%\;CI=E(IC)\pm 2\times \sqrt{V(IC)}$ (the criteria utilized in the identification of a positive safety signal: IC025 >0 (IC025: the lower bound of 95% CI));
- EBGM algorithm: $EBGM=(aN)/[(a+b)(a+c)]\;95\%\;CI=e^{\ln(EBGM)\pm 1.96\sqrt{\frac{1}{a}+\frac{1}{b}+\frac{1}{c}+\frac{1}{d}}}$ (positive safety signals require EBGM05 >2 (EBGM05: the lower bound of 95% CI)).

**Table 1:** Two-by-two table showing the variable used to compute the reporting odds ratio and proportional reporting ratio.

	Target AEs	Non-target AEs
Cyclosporine	a	b
Non-Cyclosporine	c	d

Total n=a+b+c+d; AEs: adverse events.

All data processing and statistical analyses used MYSQL 8.0, Navicat Premium 15 (https://www.navicat.com/), Excel 2019 (https://www.microsoft.com/), and GraphPad Prism 8 (GraphPad Software, USA).

## Results

### Characteristics in real world population

As depicted in Table 2, a total of 19,582 case reports related to 3,911 adverse events (AEs) were gathered between the first quarter of 2013 and the fourth quarter of 2022. Among these AEs, 476 were specifically identified as ADRs associated with cyclosporine. Furthermore, women represented 57.32% of the reported cases, while men accounted for 30.26%. Regarding age groups with specific information, individuals aged 60-79 constituted 15.04%, followed by those aged 40-59 at 12.91%. The primary contributors to the reports were the United States (8,793, 44.9%), Japan (1,607, 8.21%), Canada (1,006, 5.14%), the United Kingdom (486, 2.48%), France (325, 1.66%), and China (316, 1.61%). Among the reported serious outcomes, hospitalization (2,324, 11.87%) was the most common, followed by death (1,903, 9.72%), life-threatening events (351, 1.79%), and disability (154, 0.79%).

**Table 2:** Features of events associated with cyclosporine from the first quarter of 2013 to the fourth quarter of 2022.

	Cyclosporine	
	Counts	Percentages
Number of events	19,582	
Gender		
Male	5,925	30.26%
Female	11,224	57.32%
Unknown	2,433	12.42%
Age		
≤19	1,173	5.99%
20-39	1,295	6.61%
40-59	2,528	12.91%
60-79	2,946	15.04%
≥80	623	3.18%
Unknown	11,017	56.26%
Reporting countries (top ranked)		
United States	8,793	44.90%
Japan	1,607	8.21%
Canada	1,006	5.14%
United Kingdom	486	2.48%
France	325	1.66%
China	316	1.61%
Serious outcomes		
Hospitalization	2,324	11.87%
Death	1,903	9.72%
Life-threatening	351	1.79%
Disability	154	0.79%

### Signal detection

The signal detection reports for cyclosporine at the SOC level are presented in Table 3. According to statistics, cyclosporine-induced AEs specifically targeted 27 SOCs. The top six SOCs, ranked by the number of cases of ADRs, are as follows: eye disorders (case number: 7740), injury, poisoning, and procedural complications (case number: 4256), immune system disorders (case number: 1464), infections and infestations (case number: 1448), renal and urinary disorders (case number: 1186), and nervous system disorders (case number: 1158). All these terms are listed on the cyclosporine label. Furthermore, additional SOCs were identified but not mentioned on the label, including hepatobiliary disorders (case number: 206), pregnancy, puerperium, and perinatal conditions (case number: 205), endocrine disorders (case number: 81), and reproductive system and breast disorders (case number: 4).

**Table 3:** Signal strength of adverse events of cyclosporine at the System Organ Class (SOC) level in the FDA Adverse Event Reporting System (FAERS) source.

SOC Code	SOC	Case reports	ROR (95%CI)	PRR (95%CI)	χ2	IC (IC025)	EBGM (EBGM05)
10015919	Eye disorders	7740	33.72 (32.75-34.72)	20.79 (20.42-21.17)	141830.27	4.31 (4.22)	19.87 (19.3)
10022117	Injury, poisoning, and procedural complications	4256	5.59 (5.41-5.79)	4.59 (4.47-4.72)	12435.30	2.19 (2.08)	4.56 (4.4)
10021428	Immune system disorders	1464	14.06 (13.32-14.84)	13.08 (12.45-13.76)	15946.84	3.67 (3.49)	12.72 (12.06)
10021881	Infections and infestations	1448	10.07 (9.54-10.63)	9.4 (8.94-9.88)	10717.58	3.20 (3.02)	9.22 (8.73)
10038359	Renal and urinary disorders	1186	7.24 (6.82-7.68)	6.86 (6.49-7.25)	5898.03	2.76 (2.56)	6.77 (6.38)
10029205	Nervous system disorders	1158	7.96 (7.5-8.46)	7.55 (7.14-7.99)	6520.62	2.90 (2.69)	7.44 (7.01)
10077536	Product issues	985	27.58 (25.82-29.46)	26.24 (24.65-27.94)	22574.71	4.63 (4.41)	24.78 (23.2)
10018065	General disorders and administration site conditions	719	5.8 (5.38-6.25)	5.62 (5.23-6.04)	2712.56	2.47 (2.22)	5.56 (5.16)
10040785	Skin and subcutaneous tissue disorders	598	6.59 (6.07-7.16)	6.42 (5.93-6.95)	2710.61	2.67 (2.39)	6.34 (5.84)
10022891	Investigations	549	6.83 (6.27-7.44)	6.66 (6.13-7.24)	2612.63	2.72 (2.43)	6.58 (6.04)
10017947	Gastrointestinal disorders	443	26.45 (24.01-29.14)	25.87 (23.53-28.45)	9996.32	4.61 (4.29)	24.45 (22.19)
10005329	Blood and lymphatic system disorders	433	8.41 (7.64-9.25)	8.24 (7.5-9.06)	2710.52	3.02 (2.7)	8.1 (7.36)
10047065	Vascular disorders	236	7.5 (6.59-8.54)	7.42 (6.53-8.43)	1290.71	2.87 (2.44)	7.31 (6.42)
10019805	Hepatobiliary disorders	206	34.79 (30.17-40.13)	34.44 (29.9-39.66)	6190.30	5.0 (4.52)	31.94 (27.69)
10036585	Pregnancy, puerperium, and perinatal conditions	205	4.33 (3.77-4.97)	4.29 (3.74-4.92)	514.12	2.09 (1.63)	4.26 (3.71)
10038738	Respiratory, thoracic, and mediastinal disorders	181	9.46 (8.16-10.97)	9.38 (8.1-10.86)	1327.21	3.2 (2.71)	9.2 (7.93)
10029104	Neoplasms benign, malignant, and unspecified (incl cysts and polyps)	125	7.47 6.26-8.92)	7.43 (6.23-8.86)	684.52	2.87 (2.28)	7.32 (6.13)
10028395	Musculoskeletal and connective tissue disorders	111	7.87 (6.52-9.5)	7.83 (6.5-9.45)	650.13	2.95 (2.32)	7.71 (6.39)
10014698	Endocrine disorders	81	7.52 (6.03-9.37)	7.49 (6.02-9.33)	447.94	2.88 (2.16)	7.38 (5.92)
10007541	Cardiac disorders	64	34.41 (26.67-44.41)	34.31 (26.6-44.24)	1915.43	4.99 (4.16)	31.82 (24.66)
10042613	Surgical and medical procedures	44	60.04 (43.78-82.34)	59.9 (43.71-82.11)	2234.52	5.72 (4.69)	52.64 (38.39)
10013993	Ear and labyrinth disorders	37	25.31 (18.16-35.28)	25.27 (18.14-35.19)	814.17	4.58 (3.51)	23.91 (17.16)
10027433	Metabolism and nutrition disorders	26	5.82 (3.95-8.58)	5.82 (3.95-8.56)	102.32	2.52 (1.29)	5.75 (3.9)
10010331	Congenital, familial, and genetic disorders	18	12 (7.51-19.17)	11.99 (7.5-19.14)	176.29	3.55 (2.08)	11.68 (7.31)
10041244	Social circumstances	8	5.83 (2.9-11.71)	5.83 (2.9-11.71)	31.56	2.53 (0.46)	5.76 (2.87)
10038604	Reproductive system and breast disorders	4	31 (11.23-85.54)	30.99 (11.23-85.51)	108.23	4.86 (2.06)	28.96 (10.49)
10037175	Psychiatric disorders	3	14.21 (4.5-44.89)	14.2 (4.5-44.87)	35.64	3.78 (0.79)	13.78 (4.36)

ROR: reporting odds ratio; PRR: proportional reporting ratio; IC025: the lower bound of 95% CI; EBGM: empirical Bayes geometric mean.

The signal detection reports for cyclosporine at the PTs level are shown in Supplementary Table S1. Based on the four algorithms, a total of 476 ADR words were identified in association with the 27 SOCs at the PTs level. The top five ADRs, determined by the EBGM method, were punctal plug insertion (case number: 26, EBGM: 370.19 (129.18)), acute graft *vs* host disease in the liver (case number: 71, EBGM: 144.41 (108.46)), biliary anastomosis complication (case number: 19, EBGM: 176.43 (98.08)), scleral hyperemia (case number: 61, EBGM: 116.84 (87.01)), and acute graft *vs* host disease in the skin (case number: 148, EBGM: 98.01 (81.53)). These ADRs were associated with surgical and medical procedures, hepatobiliary disorders, injury, poisoning, procedural complications, eye disorders, and immune system disorders. With the exception of the biliary anastomosis complication, the other PTs are consistent with those in the manual. Furthermore, by examining the table of the top 40 strongest signal ADRs, a new ADR of spermatozoa decreased progressive motility was identified (case number: 7, EBGM: 63.62 (28.5)), which was associated with investigations at the SOC level.

## Discussion

Cyclosporine is an immunosuppressive drug that selectively targets T lymphocytes. It achieves this by inhibiting the production of interleukin-2 (IL-2) and the expression of the IL-2 receptor. Additionally, cyclosporine reduces the production and accumulation of cytokines, thereby alleviating the inflammatory reaction. It also improves the size and selectivity of the glomerular basement membrane, leading to a reduction in filtration and promoting the reconstruction of foot processes. These effects contribute to a decrease in urine protein concentration and the suppression of mesangial cell proliferation (17,18). Cyclosporine, employed for the prophylaxis of organ rejection in allogeneic transplants of the kidney, liver, and heart, has demonstrated expanded therapeutic applications in the management of various disorders, including dry eye disease, psoriasis, rheumatoid arthritis, systemic lupus erythematosus, atopic dermatitis, myasthenia gravis, and even during pregnancy (8,19). According to reports, cyclosporine has the ability to induce a transition towards Th2-type immune responses and cytokine release, resulting in a reduction in the Th1/Th2 ratio and an enhanced pregnancy outcome (19). As the utilization of a certain product or service broadens, there is a corresponding rise in the documentation of negative responses. Hence, it is of utmost significance to meticulously synthesize the facts pertaining to pharmaceutical utilization.

According to the FDALabel (https://www.fda.gov/science-research/bioinformatics-tools/fdalabel-full-text-search-drug-product-labeling), the primary side effects associated with the use of cyclosporine capsules therapy include renal dysfunction, tremor, hirsutism, hypertension, gum hyperplasia, glomerular capillary thrombosis, and hypomagnesemia. According to clinical studies, the following reactions occurred in 3% or greater: renal dysfunction, hypertension, cramps, skin hirsutism, acne, tremor, convulsions, headache, gum hyperplasia, diarrhea, nausea/vomiting, hepatotoxicity, abdominal discomfort, paresthesia, flushing, leukopenia, lymphoma, sinusitis, and gynecomastia. The following events occurred in approximately 2% or less of the population: allergic reactions, anemia, anorexia, confusion, conjunctivitis, edema, fever, brittle fingernails, gastritis, hearing loss, hiccups, hyperglycemia, muscular soreness, peptic ulcer, thrombocytopenia, and tinnitus. Infrequent occurrences were: anxiety, chest pain, constipation, depression, hair breakage, hematuria, joint pain, lethargy, oral ulcers, myocardial infarction, nocturnal perspiration, pancreatitis, pruritus, dysphagia, paresthesia, upper gastrointestinal bleeding, visual impairment, weakness, and weight loss. The post-marketing surveillance encompasses many adverse events, such as hepatotoxicity, heightened susceptibility to infections, headaches, and lower extremity discomfort, among others.

As women are more prone than men to suffer rheumatic immunological disorders (20), cyclosporine is mostly utilized to treat these diseases and reproductive immunity. While it is true that several significant ADs were identified that could potentially lead to substantial impairment, caution is required when interpreting the relationship between cyclosporine and these adverse events. The adverse events noted were in line with the documented safety profile of cyclosporine. However, it should be noted that several ADs were not specifically included in the medication label.

The data analysis revealed four new potential SOCs, such as hepatobiliary disorders, pregnancy, puerperium and perinatal conditions, endocrine disorders, and reproductive system and breast disorders. The liver and gallbladder are closely related organs. While cyclosporine is known for its hepatotoxicity, its effects on the gallbladder are noteworthy. Recent investigations indicated that cyclosporine can lower the ratio of Th1/Th2 (19,21), regulate the balance between Tregs and Th17 (22), and modulate maternal-fetal immune tolerance. It can also increase the proliferation and invasiveness of trophoblasts (23), reduce trophoblast oxidative stress damage and apoptosis (24), providing the immune environment required during pregnancy, improving pregnancy success rate. Thus, it is an effective treatment for immunological recurrent spontaneous abortion (RSA) patients. Although most studies have shown that cyclosporine can be taken during pregnancy with few adverse events, its pharmacological safety and effects on embryo growth and development still need additional study.

Simultaneously, there is a pressing demand for rigorous large-scale randomized double-blind controlled trials and fundamental research to delve deeper into the mechanism of action of cyclosporine and its interactions with other medications in the treatment of RSA. These investigations aim to ascertain the optimal timing, dosage, and medication cycle for the safest administration of cyclosporine, elucidate contraindications and indications, establish standardized treatment protocols, and enhance the overall clinical accessibility of this therapeutic approach.

Additionally, the top five ADRs with the strongest signal were punctal plug insertion, acute graft *vs* host disease in liver, biliary anastomosis complication, scleral hyperemia, and acute graft *vs* host disease in skin. These ADRs were associated with the SOCs of surgical and medical procedures, hepatobiliary disorders, injury, poisoning and procedural complications, eye disorders, and immune system disorders. These ADRs were all included in the cyclosporine label except the AEs of biliary anastomosis complication, which belong to the SOC of injury, and poisoning and procedural complications. Moreover, from the top 40 strongest signal ADRs table, we also found a new ADR of decreased spermatozoa progressive motility, which was associated with the SOC of investigations. These two situations are very uncommon and serious problems that are only mentioned in case reports. Due to the multiple pharmacological effects of cyclosporine, there are some cases in which cyclosporine was used beyond the instructions, such as recurrent miscarriage (25,26), recurrent implantation failure (27), and others. Therefore, we must focus more on the side effects as the scope of the drug grows in clinical practice.

Although our investigation has identified several unexpected and potential ADRs, this analysis has several limitations. Firstly, the accurate report of adverse responses may be hindered by the introduction of bias in the reporting process due to various factors, including underreporting, duplicate reports, unverified data sources, and inability to account for confounding variables. Additionally, the data analysis conducted in our study relied solely on the FAERS, without using cross-validation with other databases such as the Japanese Adverse Drug Event Report database (JADER) (28), Yellow Card Scheme (29), EudraVigilance (30), MedWatch (31), and VAERS (32). Thirdly, although data mining algorithms like EBGM can generate hypotheses, they should not be understood as incidence rates and should be used in conjunction with clinical judgment and the availability of epidemiological or clinical evidence. It is crucial to remember that, even when used in tandem, the four approaches used in this study each have their own set of intrinsic limitations. Therefore, it is possible that the findings of our research may not be consistent with those of other investigations. It is expected that advances in computer technologies will produce more precise conclusions in the future and we will keep them up to date.

### Conclusion

In this study, we utilized pharmacovigilance analysis of the FAERS database to systematically and objectively assess the potential hazards and safety signal spectrum associated with cyclosporine therapy. The updating and improvement of cyclosporine indications and contraindications are necessary in light of emerging findings. Our findings may provide guidance for future studies on cyclosporine and its medicinal use.

## Supplementary Material

Click here to view [xlsx].
